# Burn scar regeneration with the “SUFA” (Subcision and Fat Grafting) technique. A prospective clinical study

**DOI:** 10.1016/j.jpra.2018.05.001

**Published:** 2018-05-22

**Authors:** Francesco Gargano, Scott Schmidt, Peter Evangelista, Leslie Robinson-Bostom, David T. Harrington, Kristie Rossi, Yfan Guo, Paul Liu

**Affiliations:** a The Institute for Advanced Reconstruction at The Plastic Surgery Center, Sycamore Ave., Shrewsbury, NJ, United States; b Division of Plastic Surgery, The Warren Alpert Medical School of Brown University, Richmond St., Providence, RI, United States; c Department of Radiology, The Warren Alpert Medical School of Brown University, Richmond St., Providence, RI, United States; d Department of Dermatology, The Warren Alpert Medical School of Brown University, Richmond St., Providence, RI, United States; e Department of Surgery, The Warren Alpert Medical School of Brown University, Richmond St., Providence, RI, United States

**Keywords:** Pressure ulcer, Decubitus ulcers, Pressure sore treatment, Prevention of pressure ulcers, De-epithelialized flap, Pressure sore protocol

## Abstract

**Background:**

Treatment of burn scars with traditional surgical techniques is challenging due to recurrent contractures. Fat grafting has been previously used in small clinical series and results are often biased by lack of scientific validating methods. Fat grafting in clinical practice is often evaluated for its filler properties and rarely scientifically validated for its potential in dermal regeneration. Animal studies have shown dermal regeneration with new deposition and reorientation of the collagen fiber. Our study aims to apply the validity of in vitro studies to clinical practice.

**Methods:**

Our study prospectively evaluated outcomes in 12 patients treated with the “SUFA” technique (Subcision and Fat Grafting) for debilitating contracted burns scars limiting range of motion. Results were evaluated clinically with the Vancouver scale and by range of motion at 1, 3, 6 and 12 months. Dermal regeneration was evaluated by looking at dermis thickening using high definition ultrasound and scar remodeling looking at reorientation and new deposition of collagen fibers with hematoxylin-eosin histology and monoclonal antibodies against collagen type 1 and 3.

**Results:**

Statistically significant clinical improvements in range of motion of the affected joints was observed (*P*<0.05). Fat reabsorption occurred with a mean of 40%. Thickening of dermis and redistribution and reorientation of the collagen fibers within the dermis was also demonstrated.

**Conclusions:**

Our results present the first clinical scientific evidence of dermal regeneration in fat grafting. Using monoclonal antibodies and high definition ultrasounds, we demonstrate the first evidence of dermis regeneration in a clinical scenario.

## Introduction

Burn reconstructive surgery is challenging for debilitated function, cosmetic deformities and frequent lack of autologous tissue. Primary excision and grafting of deep second-degree and full-thickness burns has become the standard in most developed countries. Second and third degree heal by contraction, subsequent tension and hypertrophic scars that can be decreased by early excision and grafting.^1^

Fat grafting in burn clinical studies have been conducted on small subsets of patients, lacking scientific validation.^2^ Revascularization, decreased scarring, decreased fibrosis and increased dermis thickening with rearrangement of the collagen fibers within the dermis has been proven in murine models.^3^ Our study aims to apply the validity of in vitro studies to clinical practice.

### Material and methods

Our IRB approved study prospectively analyzed 12 patients treated with the Subcision (percutaneous release of deep scar tissues) and Fat Grafting (“SUFA”) technique. Criteria of inclusion were patients with contracted scar bands in flexion creases such as the face and neck (*n*=6), upper extremity and axilla (*n*=3), lower extremities (*n*=2) and perineum (*n*=1) All analyzed scars limited range of motion of the affected joints and had been initially treated with scar release, skin grafts, or Z-plasties performed at least 1 year from the initial insult.

Scars were evaluated clinically using the Vancouver scale. Joint range of motion was assessed using a goniometer at 1, 3, 6 and 12 months. In three patients, the dermal regeneration has been demonstrated by histology with hematoxylin-eosin and rabbit monoclonal antibodies staining for collagen type I and III before and after treatment. Fat graft survival and skin changes have been evaluated by high frequency 18MHz ultrasound.^4^ Both ultrasound and biopsy have been performed at fixed anatomic landmarks to avoid bias. Statistical significance was analyzed using the Student's t-test.

### Technique

Scar bands were positioned under tension on the operative table to facilitate its release. The abdomen represented the donor site for fat harvesting in all patients. The fat was aspirated with 60 cc syringes under negative pressure and was first washed with ringer lactate and then processed on non-adherent gauze pads. Subcision (percutaneous release) of the scar cords was performed with 18G. The “Rigottomy” technique was utilized (as described by Gino Rigotti), creating multiple tiny 2 mm cavities to release and stretch-expand contracted scars.^5^ Fat was then injected with 2.5 mm lipofilling cannulas in a three-dimensional pattern with 0.3 ml of fat injected at each pass.

Special considerations have been applied to anatomic areas. The face was injected mainly at the jaw line to minimize postoperative disfiguration in case of weight gain. On the hands, subcision occurred between dermis and extensor tendons and then fat grafted in the released space.

Postoperative protocol included oral antibiotics for three days and suture removal at 1 week. Stretching exercises were started at postoperative day 5. Between surgical sessions, an interval of 2–3 months was respected to allow graft take and remodeling. A maximum of three sessions have been employed.

## Results

Mean operative time was 70 min, mean tumescent fluid infiltrated was 100 cc and mean injected fat grafts were 45 cc.

**Figure 1:**
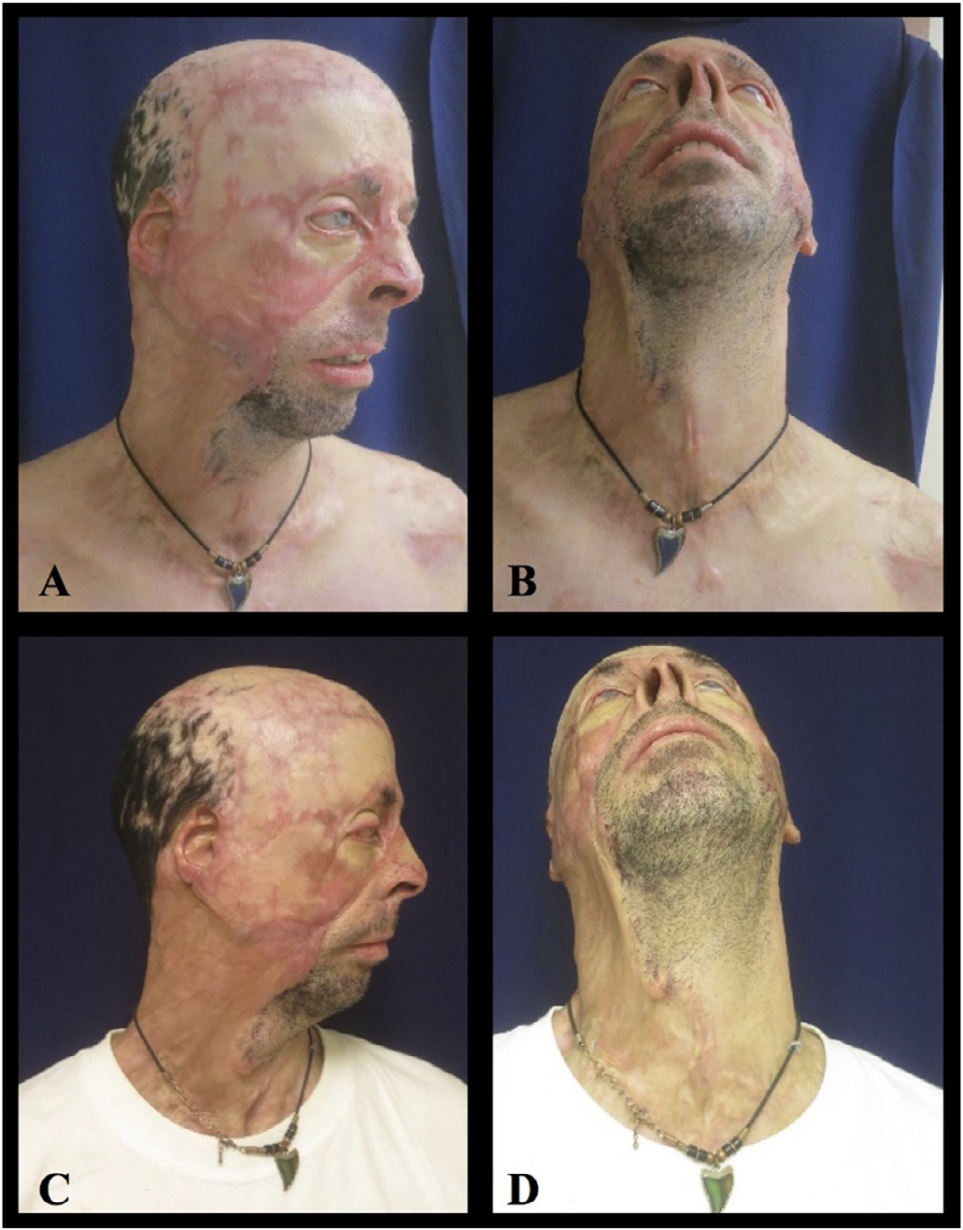
Range of motion pre-operatively (A, B) and 12 months post-operatively (C, D).

All patients showed statistically significant improvements in scar contractures in the Vancouver scale and for range of motion of the head, neck, hands, lower extremities, and perineum (*P*<0.05) (Table 1, Figure 1). High frequency ultrasound showed mean fat reabsorption of 40% and increased dermal thickness (Table 1). The histology staining with rabbit monoclonal antibodies showed increased deposition of collagen I after the fat grafting and regular orientation of the fibers as in mature healing dermis. One patient had skin breakdown resulting in uneventful healing. No donor site morbidity was noticed.

**Table 1:** 

	Pre-operative	Post-operative	*P*-value (T-test)
Vancouver scale	10 (9–12)	4 (3–7)	0.0025
Range of motion neck (rotation)	82 (50–100)	123.8 (90–160)	0.0166
		[41.8]	
Range of motion neck (flex/ext)	36.3 (20–45)	61.2 (45–70)	0.0451
		[24.9]	
18 MHz US dermal thickness	0.2 mm	0.35 mm	0.0006
Fat resorption		40% (25–60)

## Discussion

Non-surgical methods of scar release are often disappointing. Surgical corrections of contracted scars are excision and reconstruction with local tissue rearrangement such as Z-plasties, flaps, tissue expansion and skin grafting. All surgical methods carry the potential of lack of tissue, “borrowing from Peter to give Paul” and the event of a new contracture. For these reasons, we think that subcision and fat grafting represents a worthy approach to contracted scars. With remodeling of the scar from inside, the risk of a new contracture is minimized.

Limitations of this study include the small sample size and the possibility of information bias cause by measurement error when evaluating scars and range of motion. However, our study provides promising findings for the treatment of contracted scars. Compared to previous publications, our clinical outcomes show scientific validation of the “regenerative effect” of fat grafting by using high definition ultrasound and histology changes within the dermis and histology findings of rearrangement of the collagen type I fibers within the dermis.^6^ We suggest that future research on this technique should be performed using a larger sample size in a randomized clinical trial setting.

## Conclusion

The “SUFA technique” cannot be considered effective at 100% in all cases, especially when the scars are thick and extremely limiting range of motion. In the future, our study will aim to include patients in the very early stages of burns to try and modify the natural history of the retracting scars.

Our results show for the first time validation of grafting outcomes previously published only in vitro studies. Improvement in range of motion, thickening of the dermis and fat graft survival are proof of the novelty and validity of this technique in clinical scenarios.

## Conflict of interest

None of the authors have any conflicts of interest to declare in relation to this article. No funds were used to perform this study.
